# Influenza A virus: sampling of the unique shorebird habitat at Delaware Bay, USA

**DOI:** 10.1098/rsos.171420

**Published:** 2017-11-15

**Authors:** Rebecca L. Poulson, Page M. Luttrell, Morgan J. Slusher, Benjamin R. Wilcox, Lawrence J. Niles, Amanda D. Dey, Roy D. Berghaus, Scott Krauss, Robert G. Webster, David E. Stallknecht

**Affiliations:** 1 Southeastern Cooperative Wildlife Disease Study, College of Veterinary Medicine, Department of Population Health, The University of Georgia, 589 D. W. Brooks Drive, Athens, GA 30602, USA; 2 Conserve Wildlife Foundation of New Jersey, PO Box 420, Trenton, NJ 08609, USA; 3 Endangered and Nongame Species Program, New Jersey Division of Fish and Wildlife, 8747 Ferry Road, Millville, NJ 08332, USA; 4 College of Veterinary Medicine, Department of Population Health, The University of Georgia, 2200 College Station Road, Athens, GA 30602, USA; 5 Department of Infectious Diseases, St Jude Children's Research Hospital, Memphis, TN 38105, USA

**Keywords:** influenza A virus, environment, Delaware Bay, avian influenza, shorebirds

## Abstract

Delaware (DE) Bay, in the northeastern USA, has long been recognized as a hotspot for avian influenza A virus (IAV); every spring, this coastal region serves as a brief stopover site for thousands of long-distance migrating shorebirds, en route to breeding grounds in the Arctic. During these stopovers, IAV has been consistently recovered from ruddy turnstones (*Arenaria interpres*) that are likely to become infected as they feed by probing sand and cobble in search of food. In May 2010–2012, we successfully isolated 19 IAV from environmental samples (sand, *n *= 18; horseshoe crab eggs, *n *= 1) obtained from DE Bay sites. Two of these viruses were subjected to laboratory conditions similar to those in the DE Bay spring-time environment, and remained infectious for 7 days. Here, through the recovery of IAV from environmental samples, temperature monitoring at and below the sand surface and simulated laboratory trials, we provide evidence that the beach environment may enable localized transmission and short-term maintenance of IAV in this unique ecosystem.

## 1. Introduction

In avian populations, namely birds in the orders Anseriformes (ducks) and Charadriiformes (shorebirds and gulls), influenza A virus (IAV) is primarily transmitted through an indirect faecal–oral route involving faecal-contaminated water [1]. Maintenance of IAV in these populations is not only dependent on the ability of the virus to remain viable in the environment used by these avian hosts, but also on viral availability to susceptible hosts. This availability can be enhanced through specific feeding behaviours that increase viral contact with the host. Dabbling ducks, which perpetuate most haemagglutinin (HA) subtypes [1–3], employ a range of feeding techniques that include skimming surface waters and grazing underwater vegetation; when in close contact/high densities such as might be found on migratory staging areas, such feeding behaviours could lead to enhanced transmission of IAV via the faecal–oral route [4].

Tens of thousands of shorebirds (Scolopacidae) and gulls (Laridae) annually use beach habitats at Delaware (DE) Bay in May–June to feed on horseshoe crab (*Limulus polymephus*) eggs. The main Scolopacide species at this site include dunlins (*Calidris alpina*), red knots (*Calidris canutus*), ruddy turnstones (*Arenaria interpres*), sanderlings (*Calidris alba*) and semipalmated sandpipers (*Calidris pusilla*), while herring gulls (*Larus argentatus*) and laughing gulls (*L. atricilla*) are the primary Laridae species*.* Many of these birds, especially the shorebirds, rely on this energy-rich food source for refuelling during long-distance spring migration flights to breeding grounds in the Arctic [5]. At DE Bay during these stopovers, IAV has been consistently isolated from faecal and cloacal swabs collected from shorebirds and gulls; most isolations have originated from ruddy turnstones [6,7]. This convergence of IAV infection, susceptible migrating birds and horseshoe crab eggs is unlike any other ecosystem yet discovered [3,8,9]. Driven by lunar cycles and increasing water temperatures [10], spawning horseshoe crabs annually lay energy-rich eggs on the beaches, at depths of up to 20 cm [11]. Though dependent on shoreline characteristics, egg densities as high as 1.2 million per metre of New Jersey (NJ) shoreline have been reported [12]. This substantial food source is critical to the survival of migrating ruddy turnstones and other shorebirds stopping at this site [13]; as such, feeding on horseshoe crab eggs plays an important role in the complex annual ecology of IAV at this location.

In addition to pecking and turning over stones, ruddy turnstones also use a foraging behaviour that involves probing several centimetres into sand and cobble [14] in an effort to locate invertebrates, and when available, buried horseshoe crab eggs [13]. Although this feeding behaviour is not directly consistent with the water-borne transmission route suggested as important in IAV transmission in waterfowl populations [15–17], it is conceivable that IAV deposited in faeces could be protected from inactivation in a wet sand substrate and be available to feeding ruddy turnstones through their probing efforts. In infected ruddy turnstones, IAV is routinely shed in faeces; faeces could easily be covered by sand on the beach by either wave or wind action and be available to birds either probing or excavating to feed on horseshoe crab eggs.

To determine if such a scenario is plausible, we attempted to: (i) recover IAV directly from the beach environment at substrate depths to which ruddy turnstones are capable of probing; (ii) measure the temperature profiles present in this environment; and (iii) experimentally assess the persistence of these viruses at the observed temperatures within a relevant media (sand/cobble).

## 2. Material and methods

### 2.1. Beach samples

**Figure 1.**
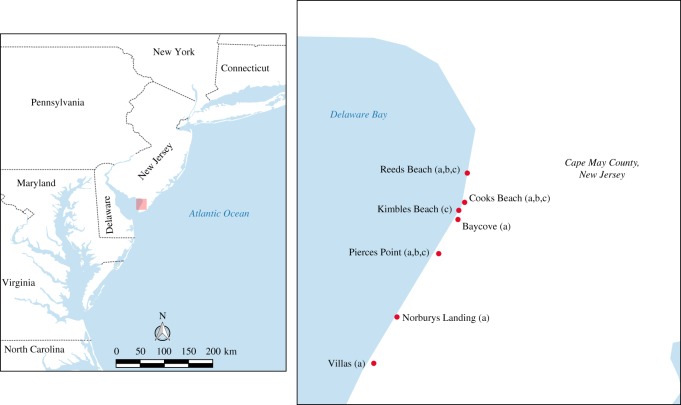
Sampling locations in New Jersey, DE Bay, USA during May 2010, 2011 and 2012 at which (*a*) IAVs were isolated from shorebird faecal or cloacal/oropharyngeal samples (2010–2012); (*b*) temperature loggers were placed (2012); and/or (*c*) environmental transects were sampled (2010–2012). Map was generated with QGIS v. 2.18.11 using Natural Earth.

Environmental samples (sand) were collected from four NJ, DE Bay beaches during 2010, 2011 and 2012 (figure 1): May 2010 at Reeds Beach (39.1244, −74.8915); May 2011 at Cooks Beach (39.1097, −74.8928) and Pierces Point (39.0839, −74.9058); and in 2012, at Pierces Point and Kimbles Beach (39.1057, −74.8957) (figure 1). For sand sampling, 3 ml syringes were modified to serve as coring devices via the removal of the bottom-most tip of the syringe. Sand was sampled in the afternoon, and on a receding tide. In 2010, 2011 and at Pierces Point in 2012, 15 m long by 4 m wide transects were divided into three zones defined as (i) backshore; (ii) high tide (HT) zone and (iii) foreshore/swash zone (at the edge of the receding tide). Approximately 20 samples, spaced 0.75 m apart, were collected in each of three transects within each zone. At the Kimbles Beach collection site in 2012, five 15 m long transects were spaced approximately 1.5 m apart from backshore to the water's edge, with 10 samples per transect, spaced approximately 1 m apart. Samples were not taken in obvious horseshoe crab depressions. Modified syringe cores were plunged into the sand/substrate to a depth of approximately 5 cm. Care was taken to avoid introducing surface sand into the sample; as such, the bottom-most 0.5 ml of sand was then dispensed into 4 ml cryovials containing 2 ml of chilled viral transport media (VTM) consisting of brain heart infusion media (Becton Dickinson and Co., Sparks, MD, USA) supplemented with antibiotics [penicillin G (1000 units ml^−1^), streptomycin (1 mg ml^−1^), kanamycin (0.5 mg ml^−1^), gentamicin (0.25 mg ml^−1^) and amphotericin B (0.025 mg ml^−1^)] (Sigma Chemical Company, St Louis, MO, USA) and the remaining volume of sand in the syringe was discarded. In 2011, opportunistic sampling of eggs in horseshoe crab excavations was carried out at Pierces Point. Small batches of freshly deposited eggs were wrapped in moistened paper towels. All samples were stored at 4°C for 24–48 h and shipped to the laboratory for storage at −80°C or immediate processing.

### 2.2. Virus isolation from environmental samples and birds

Horseshoe crab eggs were carefully removed from paper towels with sterile forceps, and two to three were placed into each of five tubes containing 2 ml of VTM. For virus isolation preparation, sand samples were vigorously vortexed for 15 s and horseshoe crab eggs were mechanically homogenized (Tissue-Tearor, Biospec Products, Inc., Bartlesville, OK, USA). Tubes containing sand or homogenized eggs were centrifuged at 1500*g* for 15 min and supernatant was inoculated (0.33 ml egg^−1^) into three 9- to 11-day-old specific-pathogen-free (SPF) embryonating chicken eggs (ECE) via the allantoic route. SPF ECE were incubated at 37°C for 120 h; amnio allanotic fluid (AAF) was then harvested and tested by haemagglutination assay using 0.5% chicken red blood cells [18]. RNA from all haemagglutination assay positive AAF was extracted using the QIAamp Viral RNA Mini Kit (Qiagen, Valencia, CA, USA) as per the manufacturer's instructions, and tested by matrix gene real-time reverse transcriptase (rrt)–PCR [19]. Identified IAV were further subtyped using HA and neuraminidase (NA) rrt–PCR specific primers as previously described [20–22]. Shorebirds were also sampled and tested for IAV by virus isolation in SPF ECE during the years of environmental sampling (2010–2012) as previously described by the University of Georgia (UGA) and collaborators at St Jude Children's Research Hospital (SJCRH) [7].

### 2.3. Temperature profiles

Temperature loggers (DS1922 L iButton, Maxim Integrated, San Jose, CA, USA) were programmed to collect temperature data every 30 min at an accuracy of ±0.5°C for approximately 48 h. Loggers were deployed on 23 May 2012 between 17. 40 and 18.40, and recovered on 25 May 2012 at 17.10. In total, 16 paired temperature loggers were placed at approximately 15 cm depth in sand/substrate and at the surface of the sand; they were attached to 30 cm long pieces of rebar with zip-ties for easy retrieval. Loggers were situated in eight locations that corresponded to HT, low tide (LT) and far low tide (far-LT) zones at Cooks Beach and Pierces Point, and at HT and LT zones at Reeds Beach (figure 1).

### 2.4. Virus persistence

The viruses used in laboratory trials were isolated in May 2012 from environmental sources (A/Sand/New Jersey/Sand2012 4-4/2012(H12N3) [Sand 4–4] and A/Sand/New Jersey/Sand2012 5-8/2012(H12N1) [Sand 5–8]) and from a ruddy turnstone faecal sample: A/Ruddy Turnstone/New Jersey/AI12-2976/2012(H12N3) [RUTU-2976]. First-passage isolates of stock viruses were propagated in SPF ECE and were stored at −80°C. Viruses were titrated on Madin Darby canine kidney cells (MDCK, American Type Culture Collection, Manassas, VA, USA) [23]; viral titres ranged from 10^6.90^ to 10^7.70^ median tissue culture infectious dose (TCID_50_) ml^−1^. Electronic supplementary material, table S1 provides a description of viruses used in this study.

Water used in the persistence trials consisted of distilled water that was buffered with 10 mM HEPES, and pH was adjusted with 1 N solutions of NaOH or HCl to provide a pH of 6.0 or 7.2. Salinity was adjusted to 20 ppt with the addition of commercially available sea salt (Morton, Chicago, IL, USA). These conditions were chosen to fall within the range of DE Bay water pH values (pH 6.0–8.5) measured in the same year in Zone 6 where sampling sites were located in our study [24] and to most closely match the average salinity of DE Bay waters in 2012 [25]. Aliquots of water were allowed to condition in a 22°C incubator, a temperature chosen based upon thermal data collected from environmental data loggers placed in the sand/substrate in DE Bay in 2012 (described above).

A large container of sand was collected from DE Bay in 2012, and was covered and stored dry at indoor ambient temperature until used in the experiment. To prepare treatments, sand was weighed and aliquoted at 25 g into 50 ml conical tubes (approximately 14.5 ml of sand per tube). Tubes of sand were placed in an environmental incubator at 22°C, while viral dilutions were prepared; stock viruses were diluted in the 22°C temperature acclimated water at between 1 : 10 and 1 : 100 to achieve a starting titre of approximately 10^5.30^ to 10^6.30^ TCID_50_ ml^−1^. Tubes of sand were then hydrated with 8 ml of the viral-inoculated water (sand + water treatment; SW); viral-inoculated water was also added to 50 ml conical tubes with no sand to serve as controls (water treatment; W). Given the granularity of the sand used, 8 ml of water was found to fully hydrate approximately the bottom 14.5 ml of sand. Experimental conditions are summarized in electronic supplementary material, table S1.

Water samples from the water (W) and sand + water (SW) treatments were collected immediately after inoculation (0 days post-inoculation (dpi)) and then at 1, 2, 3, 4, 5 and 7 dpi. For the W treatments, tubes were vortexed, and 0.70 ml water was removed. For SW treatments, a sterile 1 cc syringe with 20 G needle was inserted into each sand sample to 2.5 cm depth and approximately 0.70 ml of water was removed. At 7 dpi, a 1 ml sand core (SC) also was taken from all SW treatments and processed for virus titration and matrix rrt–PCR as previously described for beach samples. Virus titres in sand and water samples were determined by microtitre endpoint titration in MDCK cells as described [26]. Additionally, RNA from most samples was extracted as described for AAF, and quantified for the matrix gene of IAV on a SmartCycler (Cepheid, Inc., Sunnyvale, CA, USA) using primers and probe as described [27]; cycle threshold (Ct) values were recorded for all RNAs extracted.

### 2.5. Statistical analyses

Viral titres were calculated according to the method of Reed & Muench [28]. Linear regression was used to determine a 90% reduction time (Rt) for each virus/treatment combination that demonstrated more than a 1 log_10_ TCID_50_ ml^−1^ reduction in viral titre over the course of the trial; Rt values correspond to the time required for a decrease in viral titre by 1 log_10_ TCID_50_ ml^−1^. The minimum detectable limit for this procedure is 10^1.77^ TCID_50_ ml^−1^.

The effects of experimental factors on viral titres and Ct values were evaluated using linear mixed models with virus as a random effect. Models included main effects for sample type (W or SW), pH (6.0 or 7.2) and day post-inoculation (continuous), as well as all possible two-way interactions between these three variables. Normal probability plots and plots of the residuals versus the fitted values were used to assess the normality and homogeneity of variance assumptions. All tests assumed a two-sided alternative hypothesis, and *p*-values of less than 0.05 were considered statistically significant. Analyses were performed using commercially available statistical software (JMP v. PRO 12, 1989–2007; SAS Institute Inc., Cary, NC, USA).

## 3. Results

### 3.1. Natural environment

Viruses were successfully isolated from DE Bay SC samples in 2010 and 2012. In 2010, 17 H13N6 IAV viruses were isolated from 192 sand samples, with at least four recovered from every zone sampled at Reeds Beach. In 2012, no IAV were isolated from 229 sand samples collected at Pierces Point; one H12N1 and one H12N3 from 230 sand samples were isolated on different transects at Kimbles Beach. No viruses were isolated from 360 SCs collected at either Cooks Beach or Pierces Point in 2011; however, one low pathogenicity H7N3 IAV was obtained from one of five homogenized horseshoe crab egg samples collected at approximately 2 cm depth at Pierces Point in 2011. Matching HA/NA subtype combinations were also recovered from swabs or environmental samples collected from shorebirds at the same sites and in the same years by UGA and/or SJCRH (table 1).

**Table 1.** Influenza A viruses recovered from environmental samples (sand from transects in 2010 and 2012; horseshoe crab eggs in 2012) collected from three beach locations at DE Bay, USA, and matching HA/NA subtypes from shorebirds collected at DE Bay in the same year.

collection year	location	transect	strain name	subtype	recovery of matched HA/NA subtype from shorebirds^a^
2010	Reeds Beach	backshore zone#1	A/sand/NJ/Sand2010 1-17/2010	H13N6	SJCRH^b^ (1): Reeds Beach (RB)-1
			A/sand/NJ/Sand2010 1-19/2010		
			A/sand/NJ/Sand2010 1-24/2010		
			A/sand/NJ/Sand2010 1-27/2010		
		swash/high tide zone#2	A/sand/NJ/Sand2010 2-11/2010		
			A/sand/NJ/Sand2010 2-35/2010		
			A/sand/NJ/Sand2010 2-37/2010		
			A/sand/NJ/Sand2010 2-38/2010		
			A/sand/NJ/Sand2010 2-42/2010		
			A/sand/NJ/Sand2010 2-43/2010		
			A/sand/NJ/Sand2010 2-47/2010		
			A/sand/NJ/Sand2010 2-49/2010		
			A/sand/NJ/Sand2010 2-56/2010		
		foreshore zone#3	A/sand/NJ/Sand2010 3-1/2010		
			A/sand/NJ/Sand2010 3-7/2010		
			A/sand/NJ/Sand2010 3-26/2010		
			A/sand/NJ/Sand2010 3-72/2010		
2011	Pierces Point	N/A	A/horseshoe crab egg/NJ/AI11-673/2011	H7N3	UGA^c^ (7): Cooks Beach (CB)-2; Pierces Point (PP)-1; RB-3; Villas-1
					SJCRH (8): CB-4; Kimbles Beach (KB)-2; PP-2
2012	Kimbles Beach	Transect #4^d^	A/sand/NJ/Sand2012 4-4/2012	H12N3	UGA (20): Baycove (BC)-2; CB-1; KB-12; PP-4; RB-1
					SJCRH (7): Norburys Landing-1; PP-6
		Transect #5^e^	A/sand/NJ/Sand2012 5-8/2012	H12N1	UGA (14): BC-4; CB-2; KB-5; PP-3

^a^Collecting institution (total number of isolates): beach location (abbreviation) − number of isolates from location.

^b^St Jude (SJCRH) IAV isolates were obtained from faecal swabs.

^c^University of Georgia (UGA) IAV isolates were obtained from faecal swabs or cloacal/oropharyngeal swabs taken directly from shorebirds or gulls.

^d^Transect #4 was located 1.5 m behind edge of receding water.

^e^Transect #5 was located at edge of receding water.

**Figure 2.**
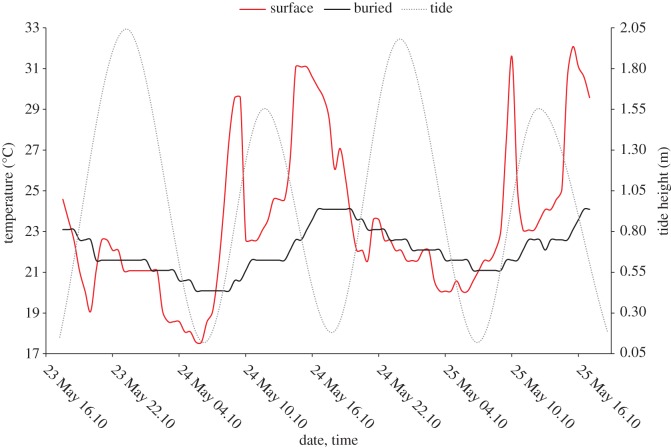
Cooks Beach HT temperature (°C) profiles for surface (solid, red) and buried (solid, black) temperature loggers over a 2-day time period in May 2012. Tide height in metres is reflected on the secondary axis (dotted, light grey).

Temperature profiles obtained from data loggers on the surface of the sand were highly variable, ranging as much as 22°C over a 48 h period as compared to only 9°C when buried at 15 cm at the same site in the same time period (Pierces Point, HT; electronic supplementary material, table S2). Temperature loggers in areas that were more frequently covered by water (those in far-LT and LT zones) were also less susceptible to temperature fluctuations than those placed in HT areas which were covered with water less often over the 2-day period (electronic supplementary material, table S1). Temperature loggers buried at 15 cm in the far-LT zone at Cooks and LT zone at Reeds Beaches were recovered at 2.5 and 10 cm depth, respectively. Shallow retrieval of loggers initially buried in the LT zone was probably a function of extreme tidal washing/erosion in areas that sustained wave action. Interestingly, the logger placed at 15 cm depth in the HT zone at Reeds Beach was recovered on the sand surface. The Reeds Beach site differs from the others surveyed here in that it is more residential with permanent inhabited structures, a steep, narrow beach and a bulkhead. The interaction of these parameters may have contributed to the displacement of the logger to the surface after just 2 days. The potential role of tidal cycles on buried and surface substrate temperature at the Cooks Beach HT zone is shown in figure 2; probes placed in the LT zone at Reeds Beach responded similarly, with the buried probe having a more constant temperature, regardless of tidal cycle (electronic supplementary material, figure S1). The temperature profiles of probes buried at 15 cm in far-LT and LT zones (and therefore under water more often) at Pierces Point did not undergo substantial temperature variation, regardless of tide height, while the buried probe in the HT zone was more responsive to changing tide (electronic supplementary material, figure S2).

### 3.2. Environmental persistence: experimental studies

Infectious virus was detected for 7 days in all water only (W) treatments (table 2). Infectious viruses were isolated from the water of sand + water (SW) treatments for a shorter duration than from W alone (to at least 5 days for all SW treatments except the shorebird isolate, RUTU-2976, at pH 6.0 (only detected to 3 days)); infectious virus was retrieved from all SCs at 7 dpi. RNA was detected from all treatments for all days. As expected, Ct values increased over the 7 days of the trial for all viruses and treatments. Environmental isolate Sand 4–4 showed an appreciable decrease in titre at 0 dpi in the SW treatment at pH 6.0 (3.80 TCID_50_ ml^−1^), as compared to the W control at pH 6.0 (5.90 TCID_50_ ml^−1^). Interestingly, despite a starting titre more than 1 log_10_ TCID_50_ ml^−1^ lower than the same virus in SW at pH 7.2, the SC sample from Sand 4–4 at 7 dpi had an Rt value approximately 2 days longer than that from the SC from the more neutral pH treatment (3.53 and 1.55 days, respectively).

**Table 2.** Viral titres reported as TCID_50_ ml^−1^ and IAV matrix gene Ct values, in brackets, for all viruses and treatments analysed for 7 days as part of the experimental laboratory persistence trial. Regression equations and Rt values were calculated for all treatments yielding at least a one log reduction in viral titre over the length of the trial.

			titre (TCID_50_ ml^−1^) [Ct values]									
			days post-inoculation (dpi) water samples									SC^f^ dpi sand
virus	treatment	pH	0	1	2	3	4	5	6^g^	7	regression equation, Rt value (days)	7
Sand 4–4	W^d^	6.0	5.90 [21.66]	5.57 [21.86]	5.55 [21.38]	5.52 [21.36]	5.44 [22.24]	5.41 [21.97]	NT^a^ [22.42]	5.49 [22.63]	NC^b^	n.a.^c^
		7.2	5.94 [21.60]	5.59 [21.6]	5.76 [21.65]	5.63 [21.89]	5.47 [21.99]	5.56 [23.02]	NT [22.48]	5.69 [22.43]	NC	n.a.
	SW^e^	6.0	3.80 [NT]	2.94 [NT]	1.91 [34.30]	2.76 [NT]	1.77 [NT]	1.77 [32.50]	NT [NT]	1.84 [38.93]	*y* = −0.28*x* + 3.24, 3.53	1.83 [36.85]
		7.2	5.17 [29.39]	3.16 [31.36]	1.96 [30.07]	2.30 [33.05]	1.77 [32.66]	1.80 [32.43]	NT [32.02]	ND^c^ [33.03]	*y* = −0.64*x* + 4.18, 1.55	1.80 [36.37]
Sand 5–8	W	6.0	6.32 [18.91]	6.10 [18.74]	6.01 [18.80]	6.10 [18.75]	5.63 [19.14]	6.10 [19.58]	NT [19.60]	6.10 [19.31]	NC	n.a.
		7.2	6.32 [19.13]	6.04 [18.85]	6.10 [18.75]	6.10 [19.23]	5.17 [19.33]	5.01 [19.54]	NT [19.90]	4.90 [19.54]	*y* = −0.23*x* + 6.36, 4.44	n.a.
	SW	6.0	4.81 [22.61]	3.44 [27.99]	4.01 [25.65]	2.90 [28.02]	2.47 [27.55]	1.93 [28.39]	NT [30.42]	ND [26.83]	*y* = −0.52*x* + 4.56, 1.91	3.88 [29.36]
		7.2	5.17 [21.71]	4.12 [26.68]	3.56 [25.59]	3.36 [26.78]	1.79 [27.95]	1.77 [28.34]	NT [27.68]	1.79 [26.73]	*y* = −0.50*x* + 4.65, 2.01	2.93 [30.27]
RUTU 2976	W	6.0	5.57 [20.41]	5.10 [20.76]	5.17 [20.82]	5.01 [20.39]	5.22 [20.81]	4.17 [21.70]	NT [23.55]	3.24 [24.60]	*y* = −0.31*x* + 5.72, 3.18	n.a.
		7.2	5.52 [20.36]	5.49 [20.54]	5.44 [20.58]	5.69 [20.25]	4.60 [20.50]	4.90 [21.33]	NT [22.14]	5.01 [21.39]	NC	n.a.
	SW	6.0	4.59 [24.00]	3.32 [28.58]	2.31 [28.01]	1.84 [28.82]	ND [29.15]	ND [31.13]	NT [31.85]	ND [30.61]	*y* = −0.98*x* + 4.43, 1.02	2.22 [31.53]
		7.2	5.00 [23.49]	3.28 [28.55]	2.47 [27.65]	1.93 [29.21]	1.79 [NT]	1.77 [30.33]	NT [29.16]	ND [29.35]	*y* = −0.64*x* + 4.21, 1.57	3.03 [31.35]

^a^NT, not tested.

^b^NC, did not meet criterion of one log reduction in viral titre.

^c^n.a., not applicable; ND, tested, but below limit of detection.

^d^W, water only treatment.

^e^SW, sand + water treatment.

^f^SC, sand core taken at 7 dpi.

^g^Titrations not performed on 6 dpi.

With respect to viral titres, there was a significant main effect of sample type (W or SW) (*p* < 0.001) and a significant interaction between sample type and day (*p* < 0.001), indicating that titres were significantly lower in the SW samples compared to the matched W controls on day 0, and the titres in the SW samples decreased significantly more quickly over time. There was no significant main effect of pH (*p* = 0.461) and no significant interaction between pH and day (*p* = 0.353), suggesting that there was no difference in titres for samples with pH 6.0 compared to those with pH 7.2. Likewise, there was no significant interaction between sample type and pH (*p* = 0.905). Results for the Ct values were consistent with those of the viral titres, with Ct values being significantly higher in the SW samples compared to the W samples on day 0 (*p* < 0.001) and increasing significantly more quickly for the SW samples over time (*p* < 0.001). There was no significant main effect of pH on the Ct values (*p* = 0.116) and no significant interaction between pH and day (*p* = 0.140). Finally, there was no significant interaction between sample type and pH in their effects on Ct values (*p* = 0.280).

## 4. Discussion

Within the DE Bay ecosystem, the foraging strategies of ruddy turnstones, coupled with their population density and propensity to amplify IAV, provide an opportunity to explore the interplay where the potential environmental persistence of IAV, host behaviour and viral transmission intersect. To be transmitted, viruses must remain infectious in the environment that exists between hosts and susceptible individuals, for an adequate duration, and in a form that will allow for transfer to new hosts. Given that ruddy turnstones probe substrate and debris in search of food, transmission of infectious virus buried by foraging efforts of other birds, by spawning of horseshoe crabs or by virus/faeces percolated into sand could be an expansion of the indirect faecal–oral route long recognized as the primary mode of transmission of avian IAV.

At least one IAV was successfully isolated from environmental (sand and/or horseshoe crab egg) samples every year from 2010 to 2012. In relation to viruses recovered from SCs, care was taken to collect samples from a depth at which foraging shorebirds could naturally probe. Surface sand was not included, in order to avoid (day of sampling) contamination from recently deposited shorebird faeces. Although the IAV subtype(s) isolated from SCs/horseshoe crab eggs matched the IAV subtypes isolated directly or indirectly (faeces) from shorebirds, it is unlikely that recently deposited virus would percolate to this depth without subsequent tidal action or through the action of ruddy turnstones excavating sites while feeding. Therefore, it is likely that some of the infectious viruses recovered were excreted at least several hours, and possibly a number of tidal cycles, earlier. Environmental samples were taken in close proximity to one another, and it is possible that viral recovery could be influenced by an aggregation of infected hosts, which was probably the case with the isolation of 17 H13N6 viruses in 2010. Because of such potential sampling artefacts, it is not prudent to extrapolate to estimates of IAV prevalence or subtype diversity from these environmental data.

Temperature profiles from buried and surface probes reveal a number of factors relevant to the environmental maintenance of IAV. First, buried temperature loggers, regardless of tidal positioning, showed a narrower range of temperature extremes, whereas those on the sand surface were influenced more by ambient air temperature and solar irradiation when not underwater, and by water temperature as the tide covered them. Also, a more constant layer of water on loggers (such as was the case at LT and far-LT zones) tended to further restrict temperature fluctuation (electronic supplementary material, figure S2). The ability of IAV to remain infectious is inversely related to increasing temperature; most IAV tend to be fairly quickly inactivated at high temperatures in both distilled [23,26,29–31] and natural [29,32–34] water studies. Modulation of the temperature in a given natural microenvironment, such as could be found below the surface of the sand, could play an important role in the ability of IAV to remain infectious. In addition to temperature, buried virus would also be protected from UV inactivation and, depending on the elevation of the beach, from desiccation. The movement of several loggers from buried to surface positions could provide evidence of a natural mechanism that may serve to increase the availability of these viruses to feeding birds; the observed sand movement may have resulted from wave, tide, current or wind action. Additionally, given their large body size and burrowing tendencies, the movements of horseshoe crabs have been shown to reactivate sediment and alter the release of eggs back onto the surface, often from depths not penetrated from wave action alone [35,36]. These results not only provide evidence of a microenvironment that is compatible with IAV persistence but also of a means for increasing the availability of these viruses to birds after deposition.

Under laboratory conditions at average water salinity (20 ppt) and sand temperature (22°C) observed at DE Bay in 2012, all three IAV (Sand 4–4, Sand 5–8 and RUTU-2976) remained viable in sand and were detectable by IAV matrix rrt–PCR for the duration of the experiment (7 dpi). When these relevant IAV were used in persistence trials, variation was seen across all treatments and viruses; this was expected based on previous results from experiments using distilled and natural water models [23,29,34,37,38]. Viral titres for all viruses at both pH levels decreased more quickly in SW samples than in W samples alone, indicating an effect related to the presence of sand. The sand used in these trials was not sterile nor chemically characterized and could have contained both biological and chemical components that could have had a detrimental effect on virus infectivity. Factors that may have been associated with the sand that potentially inactivate viruses include increased concentrations of salt(s), proteolytic enzymes [39] and microbial antagonism [40].

Infectious virus was isolated from the water portion of SW for 5–7 days with the two environmental isolates (Sand 4–4 and Sand 5–8) and between 3 and 5 days for the bird-origin RUTU-2976 isolate. All viruses were reisolated from the sand portion of all SC samples at the termination of the experiment (at 7 dpi), and in most cases, at a higher titre than those recovered from the water portion. The water portions of the SW samples were removed via syringe at all sampling points, and as such, very little particulate matter was included in the aliquots. The sand portions of these samples (day 7) were vigorously vortexed in media of a higher pH before supernatants were removed for titration and RNA extraction. Based on these results (virus not detected in water (SW) but detected in sand (SC) from the same sample), it appears that infective virus was bound or sequestered within the sand matrix. Sand 4–4 showed more than a 2 log_10_ TCID_50_ ml^−1^ reduction in viral titre when inoculated into the SW treatment at pH 6.0 (table 2), but the SC taken from this same treatment at 7 dpi had an Rt value of 3.53 days, the highest of any of the SC samples. Given that sand treatments were not homogeneous in particle size, abiotic or biotic components, nor of exact chemical composition, this may represent the case where a particular viral microcosm was quickly sequestered into a heterogeneous surrounding sand matrix and protected from the potentially detrimental effects of a low environmental pH. Factors such as aggregation [41] and adsorption onto surfaces or particles [42] can stabilize or help protect viruses from degradation; however, there is currently little information available related to the ability of IAV to adsorb to sandy substrates and other sediment types. Such binding may have facilitated the isolation of an IAV from horseshoe crab eggs. These eggs were naturally contaminated in the beach environment which further supports the potential role of environmental components (sand, cobble, detritus, food sources, faeces) as contributing to viral availability in the DE Bay system. The role of horseshoe crab eggs in sequestering infectious IAV as related to transmission to foraging hosts warrants further investigation.

Electrostatic charges can serve to bind or sequester viruses within columns of sand/sediment in laboratory and natural settings and the morphology, dimension and isoelectric point(s) (IEP) of viral particles and surface proteins play major roles in the fate of a given viral population in an environment. The IEP of IAV, which corresponds to the pH value at which the virus has neutral charge, has been shown to range between 4.0 and 7.0 [43], dependent upon purification technique and viral origin. For a given virus, the more positively charged the surrounding sediment is, the more likely the virus is to be adsorbed, whereas increasingly more alkaline pH would favour desorption of virus from sediment through the generation of strong repulsive forces [44,45]. Finally, the potential implications of the properties of AAF (in which the viruses used in our persistence trial were propagated) bear mentioning. Factors such as the pH of AAF, recorded to be between 6.6 and 7.1 at 14 days of chicken embryo development [46,47], and the varied biochemical and physical properties of its primary component albumen [48] and other proteinaceous matter, may be contributing to the tendency of these IAV to adsorb to or be repelled from the sand particles.

In the DE Bay IAV--shorebird relationship, viruses that can persist at relevant ambient temperatures and in/on natural substrates might be more readily available to susceptible shorebirds. Those viruses that can remain infectious longer under such conditions could serve to facilitate both the maintenance and transmission of IAV. Additionally, the combining of virus with sand may protect it from being dislodged by tidal washing. Our study demonstrates that (i) viable virus can be isolated from the beach environment at DE Bay during periods when shorebirds are infected with IAV, (ii) mechanisms exist related to ruddy turnstone feeding and the natural movement of sand to make these viruses available to feeding birds, and (iii) unknown mechanisms exist that appear to hold and stabilize these viruses in the sand matrix. Based on these observations, it is possible that the local beach environment may contribute to IAV transmission and short-term maintenance during these annual outbreaks. The isolation of IAV subtypes only present in shorebirds at the time of sampling, however, suggests that the beach environment does not contribute to long-term IAV maintenance at DE Bay, but the infection of shorebirds in this unique ecosystem, and the subsequent spread of IAV as a result of migration may play a role in the global maintenance of IAV.

## Supplementary Material

Figure S1

## Supplementary Material

Figure S2

## Supplementary Material

Table S1

## Supplementary Material

Table S2

## Data accessibility

A dataset supporting this article has been deposited at Dryad: (http://dx.doi.org/10.5061/dryad.69v95) [49].
