# Supplementary material for: Influenza A virus: sampling of the unique shorebird habitat at Delaware Bay, USA
Source: R Soc Open Sci. 2017 Nov 15;4(11):171420. doi: 10.1098/rsos.171420 (PMC5717699; doi:10.1098/rsos.171420)
Supplement: Figure S1 [file rsos171420supp1.pdf]

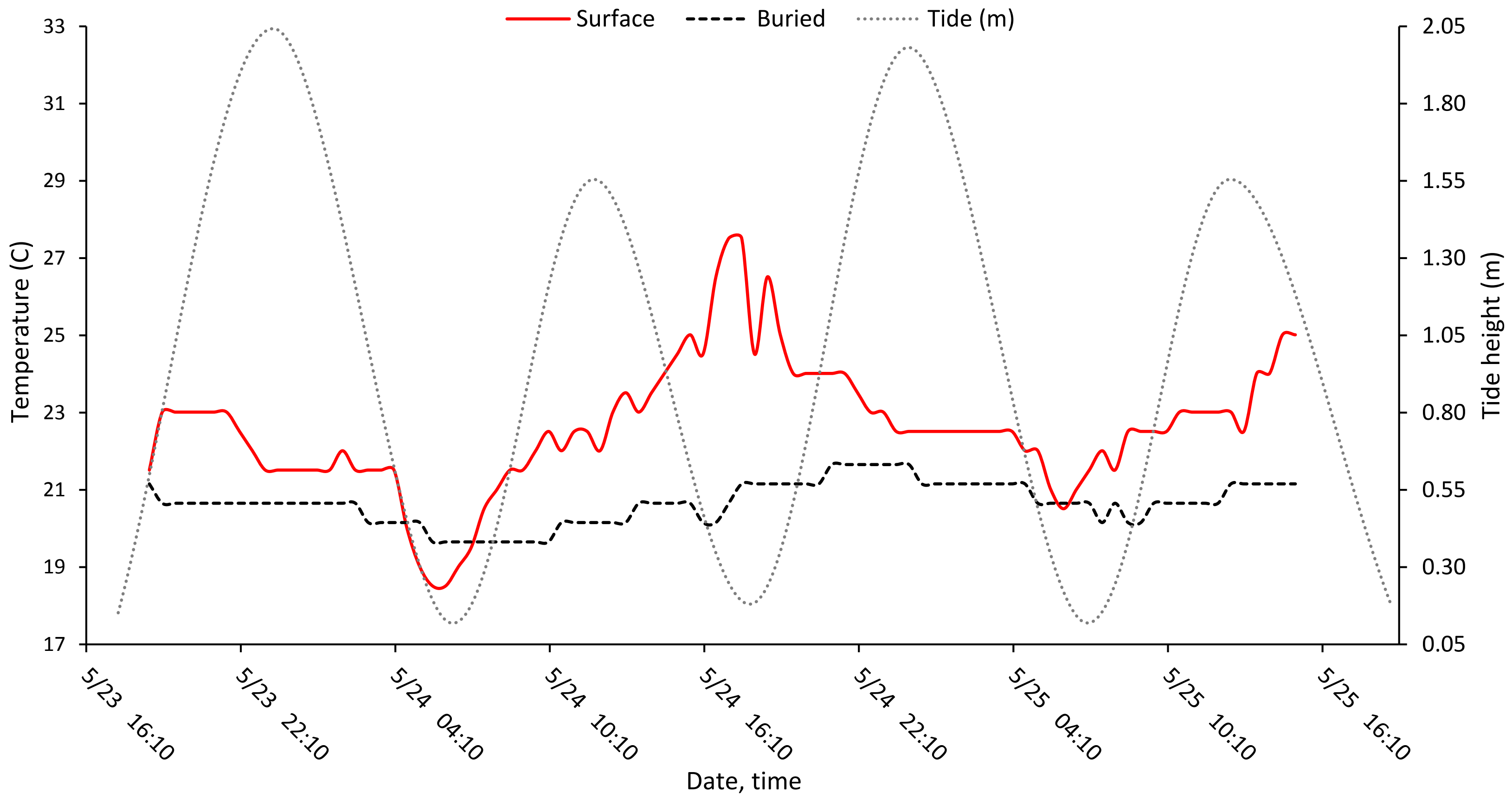

Supplemental Figure S1. Reeds Beach high tide temperature (C) profiles for surface (solid, red) and buried (dashed, black) temperature loggers over a 2-day time period in May 2012. Tide height in meters is reflected on the secondary axis (dotted, light grey).
