# Supplementary material for: Influenza A virus: sampling of the unique shorebird habitat at Delaware Bay, USA
Source: R Soc Open Sci. 2017 Nov 15;4(11):171420. doi: 10.1098/rsos.171420 (PMC5717699; doi:10.1098/rsos.171420)
Supplement: Table S1 [file rsos171420supp3.docx]

Supplemental Table S1. Description of influenza A viruses and experimental conditions used in laboratory persistence trials.

| Strain Name | ID in trial | Passage^a^ | Titer (TCID_50_/mL) | pH | Substrate^b^ |
| --- | --- | --- | --- | --- | --- |
| A/Sand/New Jersey/Sand2012 4-4/2012(H12N3) | SAND 4-4 | SPFE2 | 7.78 | 6.0 | SW, SC |
|  |  |  |  |  | W |
|  |  |  |  | 7.2 | SW, SC |
|  |  |  |  |  | W |
| A/Sand/New Jersey/Sand2012 5-8/2012(H12N1) | SAND 5-8 | SPFE2 | 7.42 | 6.0 | SW, SC |
|  |  |  |  |  | W |
|  |  |  |  | 7.2 | SW, SC |
|  |  |  |  |  | W |
| A/Ruddy Turnstone/New Jersey/AI12-2976/2012(H12N3) | RUTU-2976 | SPFE2 | 6.98 | 6.0 | SW, SC |
|  |  |  |  |  | W |
|  |  |  |  | 7.2 | SW, SC |
|  |  |  |  |  | W |

^a^SPFE2 = specific-pathogen-free embryonated chicken egg passage 2

^b^SW = sand + water; SC = sand core; W = water only
