# Supplementary material for: Influenza A virus: sampling of the unique shorebird habitat at Delaware Bay, USA
Source: R Soc Open Sci. 2017 Nov 15;4(11):171420. doi: 10.1098/rsos.171420 (PMC5717699; doi:10.1098/rsos.171420)
Supplement: Table S2 [file rsos171420supp4.docx]

Supplemental Table S2. Maximum, minimum, and mean temperatures (C) for individual temperature loggers in the beach environment, by site, tidal zone, and initial positioning on (surface) sand or buried at 15 cm.

|  |  | **Zone** | | | | | | | | |
| --- | --- | --- | --- | --- | --- | --- | --- | --- | --- | --- |
|  |  | **High Tide (HT)** | | | **Low Tide (LT)** | | | **Far Low Tide (far-LT)** | | |
| **Probe Location** | **Site** | **Max** | **Min** | **Mean** | **Max** | **Min** | **Mean** | **Max** | **Min** | **Mean** |
| **Surface** | Cooks | 32.1 | 17.6 | 23.1 | 32.1 | 17.6 | 23.4 | 27.2 | 18.2 | 22.0 |
|  | Pierces | 39.1 | 17.2 | 25.7 | 28.7 | 18.1 | 22.7 | 26.6 | 18.1 | 21.9 |
|  | Reeds | 38.7 | 17.7 | 25.4 | 27.6 | 18.6 | 26.0 | -- | -- | -- |
| **Buried (15 cm)** | Cooks | 24.1 | 20.1 | 22.1 | 21.1 | 19.1 | 20.2 | 22.6^c^ | 20.1 | 21.4 |
|  | Pierces | 28.6 | 20.1 | 23.7 | 23.6 | 19.6 | 21.7 | 22.7 | 20.2 | 21.4 |
|  | Reeds | 35.6^a^ | 19.1 | 25.4 | 21.7^b^ | 19.7 | 20.7 | -- | -- | -- |

^a^Buried probe at Reeds HT was retrieved at the surface of the sand at 48 hours

^b^Buried probe at Reeds LT was retrieved at 10cm depth at 48 hours

^c^Buried probe at Cook’s far-LT was retrieved at 2.5cm depth at 48 hours
